# SEAseq: a portable and cloud-based chromatin occupancy analysis suite

**DOI:** 10.1186/s12859-022-04588-z

**Published:** 2022-02-23

**Authors:** Modupeore O. Adetunji, Brian J. Abraham

**Affiliations:** Department of Computational Biology, St. Jude Children’s Research Hospital, Memphis, TN 38105 USA

**Keywords:** ChIP sequencing, CUT&RUN, Peak calling, Motif analysis, Cloud, Data analysis, Analysis pipeline, Computational genomics, Platform independent, GEO, SRA

## Abstract

**Background:**

Genome-wide protein-DNA binding is popularly assessed using specific antibody pulldown in Chromatin Immunoprecipitation Sequencing (ChIP-Seq) or Cleavage Under Targets and Release Using Nuclease (CUT&RUN) sequencing experiments. These technologies generate high-throughput sequencing data that necessitate the use of multiple sophisticated, computationally intensive genomic tools to make discoveries, but these genomic tools often have a high barrier to use because of computational resource constraints.

**Results:**

We present a comprehensive, infrastructure-independent, computational pipeline called SEAseq, which leverages field-standard, open-source tools for processing and analyzing ChIP-Seq/CUT&RUN data. SEAseq performs extensive analyses from the raw output of the experiment, including alignment, peak calling, motif analysis, promoters and metagene coverage profiling, peak annotation distribution, clustered/stitched peaks (e.g. super-enhancer) identification, and multiple relevant quality assessment metrics, as well as automatic interfacing with data in GEO/SRA. SEAseq enables rapid and cost-effective resource for analysis of both new and publicly available datasets as demonstrated in our comparative case studies.

**Conclusions:**

The easy-to-use and versatile design of SEAseq makes it a reliable and efficient resource for ensuring high quality analysis. Its cloud implementation enables a broad suite of analyses in environments with constrained computational resources. SEAseq is platform-independent and is aimed to be usable by everyone with or without programming skills. It is available on the cloud at https://platform.stjude.cloud/workflows/seaseq and can be locally installed from the repository at https://github.com/stjude/seaseq.

**Supplementary Information:**

The online version contains supplementary material available at 10.1186/s12859-022-04588-z.

## Background

Understanding where proteins localize on chromatin is an important component in many study designs. Selectively purifying the chromatin fragments with antibodies and sequencing the resulting material affords detection of these protein-DNA interactions. Chromatin occupancy data from ChIP-Seq or CUT&RUN-Seq experiments can support a range of biological conclusions using different analytical approaches, but each analysis usually requires intense computation and bioinformatics skills to execute. Basic processing of a chromatin binding dataset consists of several steps, including sequence read alignment to the reference genome, peak calling, annotation and visualization of peaks, coverage profiling, motif analysis, and most importantly quality control and assessment at each step. In general, the sequencing reads from a successful experiment are mapped to a reference genome, where read coverage roughly correlates with occupancy, and read-enriched regions are statistically determined. These regions or “peaks” can then be further mined to understand their functions and characteristics [1–3].

**Fig. 1:**
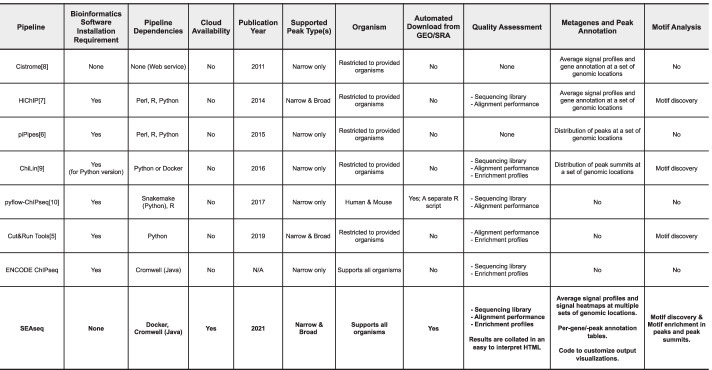
Features of the available pipelines for ChIP-seq or Cut&Run sequencing analysis

Multiple pieces of software exist for each analysis step, and attempts have been made to link these tools together in cohesive pipelines [4–10], as has been done for other analysis types [11–15]. However, these pipelines; summarized in Fig. 1, are limited by the types of analyses they can perform, often requiring substantial in-house computational infrastructure or expertise, which typically restricts their use to select research settings. Stemming from the vast number of existing tools and workflows, it remains challenging for new or seasoned researchers to decipher their usefulness and confidently determine relevant analyses.

To address these problems, we introduce a comprehensive and easy-to-use computational pipeline, “Single-End Antibody sequencing” (SEAseq), which takes advantage of enterprise-level workflow management tools to facilitate synonymous deployment across different computing infrastructures. SEAseq is a fully automated open-source pipeline that performs all the major analysis needed to process chromatin binding datasets ensuring high quality and useful results. It can be applied to data from any organism, and can automatically access publicly available data from the NCBI Gene Expression Omnibus (GEO) and Sequence Read Archive (SRA) repositories at the user’s request [16, 17]. The pipeline is designed to be platform-independent and scalable: it can be executed on a personal computer or in high-performance computing (HPC) environments. Additionally, to enable broader utility in resource-poor environments, SEAseq can also be accessed in a reliable and reasonably priced parallel cloud computing environment, available at https://platform.stjude.cloud/workflows/seaseq.

## Implementation

### SEAseq architecture

We were motivated to broaden the user base capable of performing chromatin sequencing analysis independent of computing infrastructure and expertise. To maximize the flexibility and portability of our pipeline, we adopted a workflow management system, Workflow Description Language (WDL) [18], and containerized the requisite tools, programs and SEAseq custom scripts to using Docker [19] (see Additional file 1 for the list of Docker images built for SEAseq).

The pipeline itself is not in a container platform due to its complexity; rather each step was compartmentalized into individual WDL tasks and sub-workflows to allow for independent execution of each task in an isolated environment with the use of Docker containers. Using WDL enables reorganization of individual tasks without having to redesign the entire pipeline. WDL also ensures efficient utilization and scaling of computing resources in a replicable and repeatable manner. Using Docker containers maximizes control and management of version configurations, software requirements and dependencies for improved portability and reproducibility. In addition, the choice to use WDL and Docker facilitates a standardized but customizable deployment across computing platforms, independent of the hardware infrastructure used to run SEAseq. The modular design of the pipeline allows experienced users to modify tools or steps in the pipeline, providing these modifications match the input/output schema formats used, by modifying the WDL code available on GitHub (https://github.com/stjude/seaseq).

SEAseq can be executed on a single computing node, such as a personal computer, or a parallel computing infrastructure including field-standard high-performance computing (HPC) clusters using compatible workflow execution engines such as Cromwell [20]. Complete usage instructions are outlined in the SEAseq documentation (https://github.com/stjude/seaseq/#readme). Three dependencies are required to run SEAseq: Java, Cromwell and an engine able to run Docker containers (such as Docker, Singularity [21]). Each of these packages can be installed with minimal user expertise.

### SEAseq functionality

**Fig. 2:**
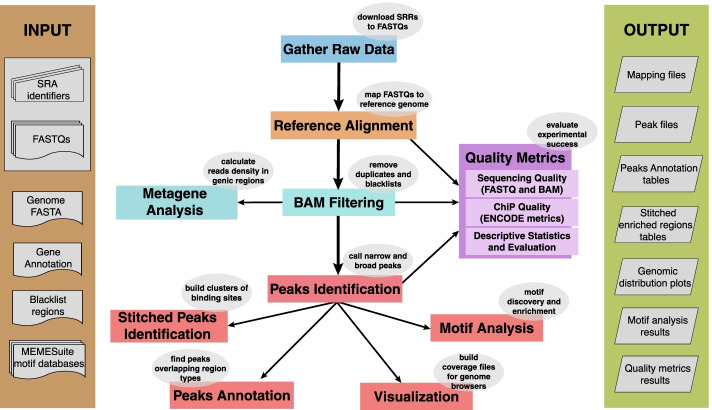
High-level SEAseq schematic. The flowchart shows the input files (left), a top-down overview of the analysis steps executed by SEAseq (center), and the outputs (right). The input consists of the specified files needed to utilize SEAseq; these files include FASTQ files (in compressed gzip [.gz] format) and/or SRA identifiers (SRR), Genome FASTA [.fa], Gene Annotation [.gtf], and optionally UHS/ DER/ DAC blacklist regions [.bed] and one or more of the MEME suite position weight matrix databases. The output consists of the analysis results files generated from SEAseq: mapping files [.bam], peaks [.bed] and peak coverage files [.wig;.tdf;.bw], per-promoter and metagene average coverage and heatmaps plots, peak annotation distribution tables, motif discovery and enrichment results, and quality metrics results [.html]

SEAseq performs the several fundamental ChIP-Seq or CUT&RUN analyses in a single execution. Figure 2 shows a schematic overview of the analyses performed by SEAseq, which are briefly described in the following paragraphs (see Additional file 2 for the expanded list of SEAseq pipeline steps and parameters).

FASTQ sequencing reads are stringently aligned to the reference genome provided using Bowtie [22]. The mapped reads are then further processed by removal of redundant reads [23] using SAMtools [24], and removal of reads in problematic regions or regions with significant background noise or artificially high signal [25] using BEDTools [26]. In addition, SEAseq characterizes the global binding preferences of the antibody using read density profiling in relevant genomic regions such as promoters and gene bodies using our custom version of BAMToGFF (https://github.com/stjude/BAM2GFF).

To compensate for the background noise intrinsic to chromatin binding assays, many analyses rely on identification of highly covered regions or peaks [3]. It is important to choose the appropriate peak-calling algorithm based on the type of protein targeted [27]. After read alignment and filtering steps are completed, SEAseq identifies enriched regions for two binding profiles: MACS [28] for factors that bind shorter regions, e.g. many sequence-specific transcription factors, and SICER [29] for broad regions of enrichment, e.g. some histone modifications. The choice of these peak callers for the identification of narrow peaks and broad peaks was based on extensive review of published benchmarking evaluations and our own previous work [30–34]. Normalized and unnormalized coverage files are generated for visualization on multiple genome browsers such as the UCSC genome browser [35] and IGV [36]. The pipeline also identifies stitched clusters of enriched regions and separates exceptionally signal-rich regions, e.g. super-enhancers, from typical enhancers using ROSE [37, 38]. SEAseq also performs motif discovery and enrichment analysis to characterize overrepresented sequences using tools from the MEME Suite [39], and performs genic annotation of the various peaks and quantifies regions of abundance in promoters, gene bodies, gene-centric windows, and proximal genes using BEDTools and custom Python scripts.

### SEAseq quality metrics and dashboard

**Fig. 3:**
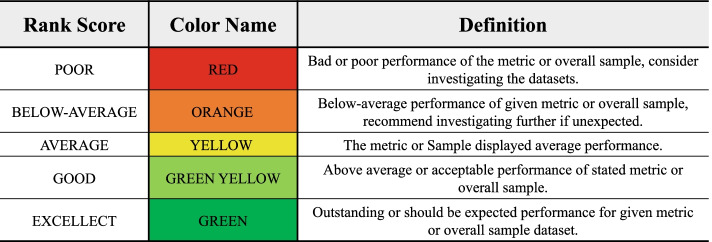
The Rank Score scheme for the SEAseq quality metrics. The rank score is flagged in a color scale for easy interpretation of metrics performance

Though other pipelines perform coverage-based analyses, most generally lack a comprehensive means of assessing the overall quality of the experiment. SEAseq uniquely calculates an extensive set of quality metrics for detecting experimental issues, including ChIP-Seq metrics recommended by the ENCODE consortium [40]. The SEAseq quality metrics include the percentage of reads mapped, nonredundant fraction (NRF), fraction of reads in peaks (FRiP), strand correlation scores (NSC, RSC), library complexity (PCR bottleneck) and many other important metrics used to infer quality as listed in Table 1. To facilitate integration and easy interpretation of these metrics, we devised a five-scale color-rank flag system to visually inspect the performance of each metric, and provide a cross-metric averaged rank score to easily intuit the performance of the overall analysis (Fig. 3). The rank flag system is estimated based on recommended thresholds from the ENCODE consortium, literature review and criteria provided in Table 2. These quality metrics can be broadly grouped into two subsets: quality assessment on the sequencing library and assessment on the enrichment profiles observed, providing users an easy way to intuitively explore the performance or quality of their data. The results are exported in a tab-delimited file and color flagged in html format. Additional file 3 shows a typical quality statistics report produced by SEAseq.

**Table 1:** SEAseq quality metrics performed and their definitions

Quality metric	Definition
Aligned percent	Percentage of mapped reads
Base quality	Per-base sequence quality distribution
Estimated fragment width	Average fragment size of the peak distribution
Estimated tag length	Sequencing read length
Fraction of reads in peaks (FRiP)	The fraction of reads within coverage-enriched regions
Linear stitched peaks (enhancers)	Total number of clustered enriched regions
Non-redundant fraction (NRF)	Fraction of uniquely mapped sequencing reads
Normalized peaks^a^	Peaks identified after input/control correction
Normalized strand-correlation coefficient (NSC)	The ratio of the maximum cross-correlation value divided by the background cross-correction
Sequence diversity	Sequence overrepresentation; if reads/sequences are overrepresented in the library
PCR bottleneck coefficient (PBC)	It is a measure of library complexity determined by the fraction of genomic locations with exactly one unique read versus those covered by at least one unique read
Peaks	Total number of enriched regions (peaks)
Raw reads	Total number of sequencing reads
Read length^b^	Average FASTQ read length
Relative strand-correlation coefficient (RSC)	A strand cross-correlation ratio between the fragment-length cross-correlation and the read-length peak
SE-like enriched regions (super enhancers)	Total number of SE-like clustered enriched regions
Overall quality	Average score rank of all metrics calculated

^a^Applicable when input/control is provided

^b^Applicable if multiple FASTQs are inputted

**Table 2:** Description of each quality metric color-rank scheme

Quality metrics	Rank				
	Excellent	Good	Average	Below-average	Poor
Aligned percent ($A$)	$A \ge 80\%$	$A \ge 70\%$	$A \ge 60\%$	$A \ge 50\%$	$A < 50\%$
Base quality ($B$)	*B *= “pass”	-	*B *= “warn”	-	*B *= “fail”
Estimated fragment width	–	–	–	–	–
Estimated tag length (E)^a^	$E < \pm 10$	–	–	–	$E > \pm 10$
FRiP ($F$)^c^	$F \ge 0.05$	$F \ge 0.02$	$F \ge 0.01$	$F \ge 0.0075$	$F < 0.0075$
Linear stitched peaks ($L$)	$L \ge 10000$	$L \ge 5000$	$L \ge 2000$	$L \ge 1000$	$L < 1000$
NRF	$NRF \ge 0.8$	$NRF \ge 0.7$	$NRF \ge 0.6$	$NRF \ge 0.5$	$NRF < 0.5$
Normalized Peaks (N)	$N \ge 10000$	$N \ge 5000$	$N \ge 2000$	$N \ge 1000$	$N < 1000$
NSC^c^	$NSC \ge 1.045$	–	–	–	$NSC < 1.045$
Sequence diversity ($D$)	*D *= “pass”	–	*D *= “warn”	–	*D *= “fail”
PBC ($C$)^c^	$C \ge 0.9$	$C \ge 0.75$	$C \ge 0.66$	$C \ge 0.5$	$C < 0.5$
Peaks ($P$)	$P \ge 10000$	$P \ge 5000$	$P \ge 2000$	$P \ge 1000$	$P < 1000$
Raw reads ($R$)	$R \ge 30 M$	$R \ge 25 M$	$R \ge 20 M$	$R \ge 15 M$	$R < 15 M$
Read length	–	–	–	–	–
RSC^c^	$RSC \ge 1$	$RSC \ge 0.75$	–	–	$RSC < 0.75$
Super stitched peaks ($S$﻿)^b^	$S \ge 0.2$	$S \ge 0.1$	$S \ge 0.05$	$S \ge 0.02$	$S < 0.02$
Overall quality (Q)	$Q \ge 2$	$Q \ge 1$	$Q \ge 0$	$Q \ge - 1$	$Q < - 1$

*M* millionreads

^a^*E* is difference between predicted tag length and average read length

^b^*S* is the ratio of superstitched regions divided by the linear stitched regions identified

^c^Extrapolated based on ENCODE recommended thresholds

### SEAseq on the cloud

To facilitate the broadest usage of our pipeline, and to empower researchers with little to no computational skills or resources, we offer a cloud-based version of SEAseq, called SEAseq Cloud, that is hosted on the St. Jude Cloud Genomics Platform [41]. The St. Jude Cloud Genomics Platform leverages Microsoft Azure and DNAnexus (https://www.dnanexus.com) to provide a secure and privacy compliant framework for analysis, storage and distribution of genomic data [41]. The DNAnexus platform provides an easy-to-navigate graphical user interface for exploration and analysis of user data, which can be quickly and securely uploaded, downloaded, and shared with collaborators at a reasonably low cost. For more advanced operations, DNAnexus also provides a command-line client. SEAseq cloud is available from https://platform.stjude.cloud/workflows/seaseq.

**Fig. 4:**
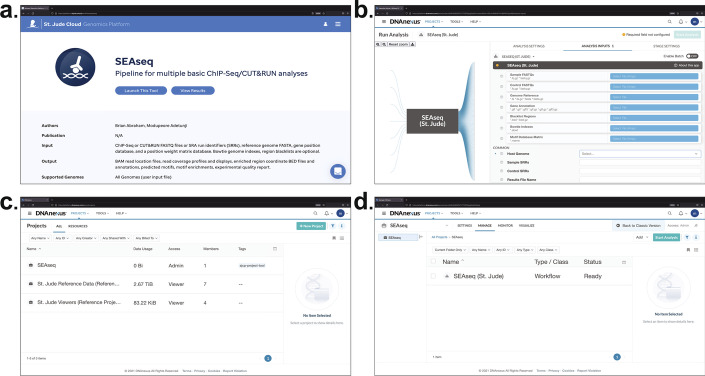
SEAseq Cloud. **a** Screenshot of SEAseq workflow Landing Page. **b** SEAseq cloud Run Analysis “Analysis Inputs” Page. **c** DNAnexus Projects Page containing SEAseq Project. **d** SEAseq cloud Project page consisting of the SEAseq workflow and where user data files can be uploaded

The user needs a DNAnexus account to use SEAseq Cloud. SEAseq requires that the user data, such as the FASTQs and genome files, be uploaded to a project folder. Navigating the SEAseq Cloud web interface after logging in, as shown in Fig. 4, involves selecting the “*Start*” button for first-time users, then the “*Launch This Tool*” button (Fig. 4a). The user will then be navigated to the SEAseq Cloud *Run Analysis* Page to start an analysis (Fig. 4b). DNAnexus requires the user input data files, such as the FASTQ files and required genome files (if applicable), be uploaded to a Project before proceeding. Uploading data files can be done on a Projects page by selecting the *Projects* tab then the *All Projects* option to be directed to the Projects/Home page (Fig. 4c). The DNAnexus Projects page (also accessible from https://platform.dnanexus.com) will contain the vended SEAseq workflow in its self-titled project and St. Jude Reference Project files (Fig. 4c). The St. Jude Reference Project files contains some genome files acceptable by SEAseq for the user’s convenience. To begin analysis, select on the SEAseq Project to access the SEAseq workflow (Fig. 4d) and/or to upload user data files by clicking the “*Add*” button. It is recommended to use the New (also called the “Pannexin”) User Interface (UI) version, instead of the Classic/Legacy (“Membrane”) version. Further descriptions will be based on the New UI as shown in Fig. 4b, d.

To start an analysis using SEAseq, select the “*SEAseq (St. Jude)*” workflow, and the user will be navigated to the *Run Analysis* page (Fig. 4b), where one can specify their preferred “*Execution Name*” and “*Execution Output Folder*” in the “*Analysis Settings*” input fields. The previously uploaded user data will then be available for selection in the requested “*Analysis Inputs*” input fields. The required fields are the sequencing FASTQs or SRA accession numbers (SRRs), “*Genome Reference*” and “*Gene Annotation*” files. SEAseq also provides a list of genome files, “*Host Genomes*”, to select from (Fig. 4b). Optionally, one or more SRRs can be inputted into the “*Sample SRRs*” field for sample SRRs and/or the “*Control SRRs*” field for Input/Control SRRs. After the analysis inputs and settings has been specified to the user’s preference, the SEAseq analysis can be started by selecting “*Start Analysis*”. Once started, the status of the job can be monitored from the project folder under the “*Monitor*” tab (Fig. 4d) or SEAseq landing page by selecting the “*View Results*” button (Fig. 4a). When the analysis is completed, a notification message will be sent to the user’s registered email and the analysis results files can be viewed and downloaded from the initially specified “*Execution Output Folder*”. More information on SEAseq Inputs and Output directories and files can be found in Additional file 4.

## Results and discussion

### Replication of two ChIP-Seq studies using SEAseq

We re-analyzed ChIP-Seq data from two previously published studies to showcase the utility of SEAseq in performing relevant analyses in a single execution.

**Fig. 5:**
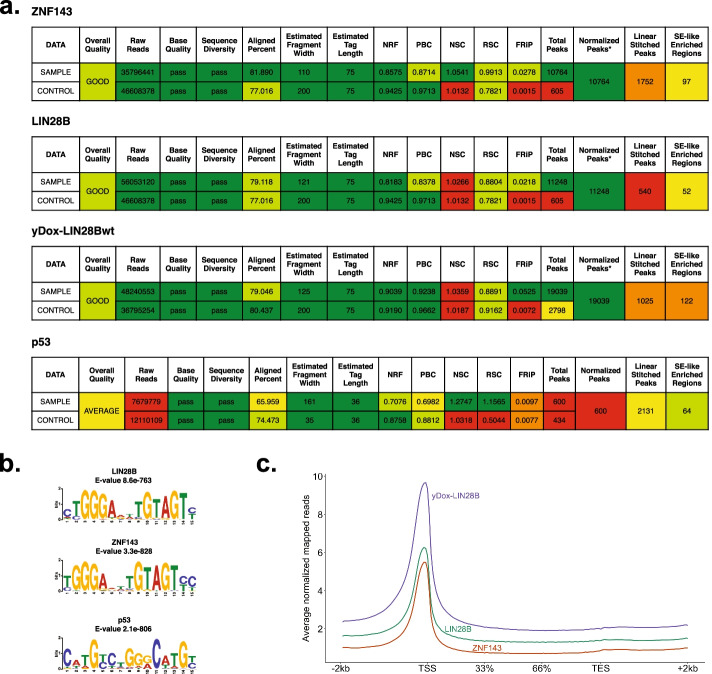
SEAseq case study. **a** Screenshot of the SEAseq Quality Evaluation Report for all studies. **b** Sequence logo depicting the top predicted motif identified for each study. **c** Genome-wide occupancy analysis of LIN28B and ZNF143 reveal enriched binding at active promoters

The first study probed functions of LIN28B in neuroblastoma and discovered it binds chromatin via interaction with the transcription factor ZNF143 (GEO accession: GSE138742) [42]. The dataset included LIN28B, ZINF143, and doxycycline-inducible engineered LIN28B ChIP-Seq data of BE2C cells. The analysis was executed using the SEAseq Cloud workflow. All required hg19 genome files and position weight matrices were first uploaded into a DNAnexus project. Each ChIP antibody SRR along with the corresponding Input DNA SRR were used as input, and the relevant genome files were selected to their required fields in SEAseq (see Additional file 5 for the accession numbers and genome files used). Analysis using SEAseq demonstrated the expected satisfactory overall scores for these samples’ read quality and quality of peaks (> 1) (Fig. 5a). Motif analysis identified the major binding consensus motif in both LIN28B and ZNF143 as reported in the original publication (Fig. 5b). In addition, given the ability of SEAseq to perform a large array of advanced downstream analysis, we also observed the expected genome-wide occupancy results, demonstrating preferential binding at promoter regions by LIN28B, ZNF143, and significantly with the doxycycline-induced LIN28B (Fig. 5e). The entire analysis of all three datasets was computed using the SEAseq Cloud platform, which successfully completed in an average of 28.45 wall clock hours costing an average of $24.14. Table 3 summarizes the total time and cost of analysis performed using SEAseq Cloud.

**Table 3:** SEAseq Cloud performance results

ChIP	Number of reads in sample	Number of reads in input	Runtime (HH:MM)	Cost
LIN28B	54,894,988	45,603,300	32:07	$26.38
ZNF143	35,108,844	45,603,300	25:49	$22.35
yDox-LIN28B	47,203,903	35,682,203	28:20	$23.68
p53	7,473,100	11,760,480	10:58	$8.33

The second study profiled binding of the tumor suppressor p53 in normal human cells (GEO accession: GSE31558) [43]. To maximize comparability with the published results, we uploaded available hg18 reference genome data files, and the deposited study files, which include ChIP-Seq for p53 in IMR90 fibroblasts (see Additional file 5). SEAseq analysis showed an “AVERAGE” overall quality score; indicative from the sequencing depth, FRiP and the corresponding poor number of peaks identified (Fig. 5a). From this, we show that the analysis may benefit from higher sequencing coverage and depth. Nevertheless, we were able to obtain comparable results to the original publication, including the significantly enriched p53 binding motif (Fig. 5b). This analysis was successfully completed in 11 h costing $8.33 (Table 3).

These recapitulated results demonstrate the utility of SEAseq in analysis of antibody purification data.

### SEAseq performance evaluation

**Fig. 6:**
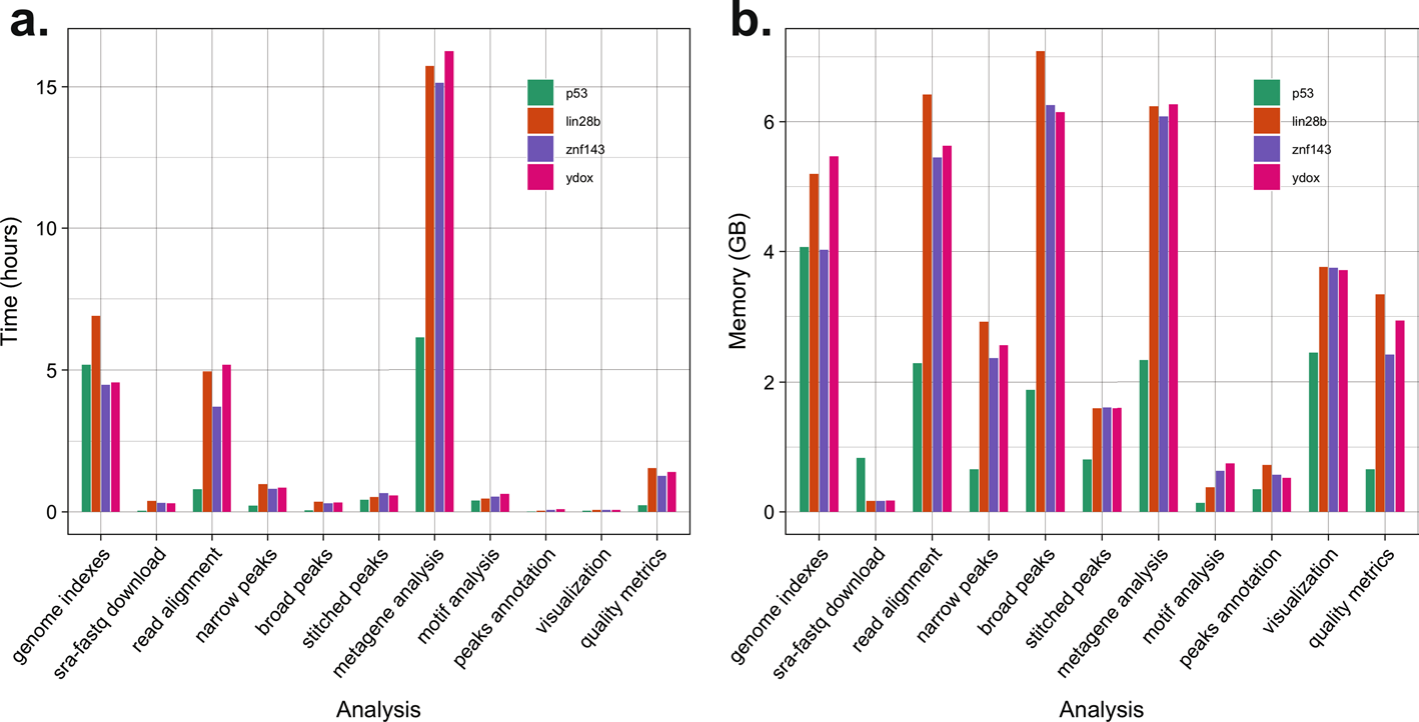
SEAseq performance results. **a** Time elapsed in hours by analysis step. **b** Memory consumption in Gigabytes (GB) by analysis step

To assess SEAseq performance and resource consumption, the aforementioned datasets using SEAseq Cloud were also analyzed on our IBM Spectrum LSF (v10.1.0.9) HPC cluster with Cromwell (v52) and Singularity (v3.8.0). Per-task analysis revealed the most time-consuming component of the pipeline is the metagene analysis step (completed in under 16 h). Most other steps were completed in under 1 h, with the exception of the read alignment and optional genome index construction steps, which each completed in 4–6 h. Figure 6 shows the time and memory consumption of the different steps. In addition, steps displaying the highest memory consumption were the metagene analysis, broad peaks identification, alignment, and genome index construction. Given the genome index construction step is computationally intensive, we recommend that users avoid this optional step by providing the bowtie genome indexes when analyzing multiple datasets. Overall, the different steps show a maximum memory usage of under 7 GB. This performance showcases the efficiency of SEAseq in appropriating tasks in a timely and memory efficient manner, and thus makes the pipeline deployable on most computing environments. Furthermore, the analysis also reveals the size of the FASTQs has a proportional effect on time and memory consumption.

## Conclusion

To our knowledge, SEAseq is the first portable, all-inclusive analysis pipeline for ChIP-Seq and CUT&RUN data. SEAseq is easy to use and provides a modular architecture for quick integration and customization for efficient data analysis on multiple computing infrastructures. Having proved highly comparable results to published datasets, we believe SEAseq fulfills a critical requirement as an efficient and reliable one-stop computational pipeline for high quality analysis results.

## Availability and requirements

- Project name: SEAseq
- Project home page: https://github.com/stjude/seaseq
- Project cloud page: https://platform.stjude.cloud/workflows/seaseq
- Operating system(s): Platform independent
- Programming language: WDL, Docker
- Other requirements: Java, Cromwell (when using the GitHub version)
- License: Apache License 2.0
- Any restrictions to use by non-academics: None

## Supplementary Information

**Additional file 1**. SEAseq Docker images. List of Docker images built for SEAseq.

**Additional file 2**. SEAseq pipeline steps and parameters. Detailed description of SEAseq pipeline steps and parameters.

**Additional file 3**. Case study LIN28B. The complete HTML quality statistics report for LIN28B ChIP-seq analysis.

**Additional file 4**. SEAseq inputs and outputs. Input files used for SEAseq analysis and detailed descriptions of the Output directories and files generated using SEAseq.

**Additional file 5**. Case study datasets. Case Study Datasets and Genome Files information.

## Data Availability

The developed software is freely available on GitHub at https://github.com/stjude/seaseq. All datasets and genome files analyzed during this study are publicly available and information on how they were obtained are included in this published article (Additional file 5).

## Abbreviations

GEO: Gene Expression Omnibus
SRA: Sequence Read Archive
WDL: Workflow Description Language
UI: User Interface
HPC: High-Performance Computing
