# Supplementary material for: SEAseq: a portable and cloud-based chromatin occupancy analysis suite
Source: BMC Bioinformatics. 2022 Feb 23;23:77. doi: 10.1186/s12859-022-04588-z (PMC8864840; doi:10.1186/s12859-022-04588-z)
Supplement: Supplementary file 2 — Additional file 2. SEAseq pipeline steps and parameters. Detailed description of SEAseq pipeline steps and parameters. [file 12859_2022_4588_MOESM2_ESM.doc]

## **SEAseq Pipeline Steps and Parameters**

SEAseq performs the following functions as shown in the Figure 2.

- If provided, the GEO/SRA identifiers (SRRs) are downloaded as FASTQs using the SRA Toolkit (<http://www.ncbi.nlm.nih.gov/books/NBK158900/>) with default parameters. N.B. paired-end data will not be processed correctly unless preprocessed into separate single-end files.
- The sequence reads FASTQ are aligned to the reference genome using Bowtie version 1.2.3 [1].

$

$ # Bowtie parameters

$ bowtie -l <fastq average readlength> -k 2 -m 2 -–best -S

$

- Mapped reads are further processed by removal of redundant reads using SAMtools v1.9 [2] and blacklisted regions with bedtools v2.25.0 [3]

$

$ # Removal of redundant reads parameters

$ samtools markdup -r -s <input.bam> <outputname>.rmdup.bam

$

$ # Removal of blacklisted regions parameters

$ intersectBed -v -a <input.bam> -b <provided blacklist.bed> > <outputname>.bklist.bam

$

- Metagene analysis in relevant genomic regions using custom version of BamToGFF v1.2.1 (<https://github.com/stjude/BAM2GFF>). SEAseq estimates the read density levels of enriched regions in the important genomic regions with respect to the whole genome. The genomic regions calculated are the promoters and gene bodies (3’UTRs, 5’UTRs, coding exons and introns). To do this, the BamToGFF tool computes the read densities of the genomic features in a user-defined range (
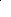
2kb from the transcription start site (TSS) and transcription termination site (TTS) by default) creating a normalized density matrix for the different genomic features. After which we generate average density plots and heatmaps of these regions, and a customizable R script will also be provided in the results directory for additional personalization if needed.
- Identification of enriched regions for two binding profiles:
  - To capture binding of factors that bind shorter regions, e.g. many sequence-specific transcription factors such as CTCF. SEAseq performs three different peak calls using MACS v1.4.2 [4].

$

$ # MACS parameters (for peaks identified excluding duplicates):

$ macs14 -p 1e-9 –keep-dup=auto –space=50 -w -S

$

$ # MACS parameters (for peaks identified keeping duplicate tags):

$ macs14 -p 1e-9 –keep-dup=all –space=50 -w -S

$

$ # MACS parameters (using a defined shift size):

$ macs14 –nomodel -shiftsize=200 –space=50 -w -S

$

- - For broad regions of enrichment, e.g. some histone modifications such as H3K27Ac using SICER v1.1.0 [5].

$

$ # SICER parameters:

$ sicer -rt 1 -w 200 -f 150 -egf 0.86 -g 200 -e 100

$

- Normalized and unnormalized coverage files generated also generated for all peaks. These coverage files (initially in wiggle format) are normalized and converted to other graphical formats such as bigwig using the wigtobigwig tool [6] and TDF format using igvtools (www.broadinstitute.org/igv/igvtools) for display on various genome browsers such as GenomePaint [7], UCSC genome browser [8] and IGV [9].
- Identification of stitched clusters of enriched regions and separates exceptionally large regions, e.g. super-enhancers from typical enhancers, using our custom ROSE program [10, 11], tagged as v1.3.0 (<https://github.com/stjude/rose>).
- Motif discovery and enrichment of enriched regions and 50bp up- and down-stream of its’ summit using the AME and MEME-chip tools respectively from the MEME Suite v5.3.3 [12].
- Annotation and Quantification of narrow and broad peaks identified in relevant genomic regions relative to the whole genome are also estimated using custom scripts. Genomic regions annotated are the promoters (
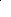
1kb from the TSS), gene-bodies (1kb upstream of the TSS to TTS), gene-centric windows (10kb upstream of the TSS to 3kb downstream of the TTS), and near proximal genes. The annotation files generated are:
  - ***centerofpeaks_closest.regions.txt***: genes nearest the center of peaks.
  - ***peaks_within_genebody.regions.txt***: peaks within gene-bodies.
  - ***peaks_within_promoter.regions.txt***: peaks within promoter regions.
  - ***peaks_within_window.regions.txt***: peaks within gene-centric windows.
  - ***peaks_compared_regions.peaks.txt***: peaks within the annotated genomic regions.
  - ***peaks_compared_regions.genes.txt***: gene information for the annotated genomic regions.
  - ***peaks_compared_regions.distribution.pdf***: percentage of peaks identified within the annotated genomic regions.
- Assessment of quality by calculating relevant metrics for detecting experimental issues or surveying the data including those recommended by the ENCODE consortium [13] using FastQC v0.11.9 , MACS v1.4.2 , SPP v1.16.0 and custom scripts. We incorporated a five-scale color-rank flag system for visual understanding the performance of each metric (Table 2,3). The flag system is based on estimated based on recommended thresholds from the ENCODE consortium, literature review and expertise.

## **References**

1. Langmead B, Trapnell C, Pop M, Salzberg SL. Ultrafast and memory-efficient alignment of short DNA sequences to the human genome. Genome Biol. 2009;10:R25. doi:10.1186/gb-2009-10-3-r25.

2. Li H, Handsaker B, Wysoker A, Fennell T, Ruan J, Homer N, et al. The Sequence Alignment/Map format and SAMtools. Bioinformatics. 2009;25:2078–9.

3. Quinlan AR, Hall IM. BEDTools: A flexible suite of utilities for comparing genomic features. Bioinformatics. 2010;26:841–2. doi:10.1093/bioinformatics/btq033.

4. Zhang Y, Liu T, Meyer CA, Eeckhoute J, Johnson DS, Bernstein BE, et al. Model-based analysis of ChIP-Seq (MACS). Genome Biol. 2008;9. doi:10.1186/gb-2008-9-9-r137.

5. Zang C, Schones DE, Zeng C, Cui K, Zhao K, Peng W. A clustering approach for identification of enriched domains from histone modification ChIP-Seq data. Bioinformatics. 2009;25:1952–8. doi:10.1093/bioinformatics/btp340.

6. Kent WJ, Zweig AS, Barber G, Hinrichs AS, Karolchik D. BigWig and BigBed: Enabling browsing of large distributed datasets. Bioinformatics. 2010;26:2204–7. doi:10.1093/bioinformatics/btq351.

7. Zhou X, Wang J, Patel J, Valentine M, Shao Y, Newman S, et al. Exploration of Coding and Non-coding Variants in Cancer Using GenomePaint. Cancer Cell. 2021;39:83-95.e4.

8. Kuhn RM, Haussler D, James Kent W. The UCSC genome browser and associated tools. Brief Bioinform. 2013;14:144–61. doi:10.1093/bib/bbs038.

9. Thorvaldsdóttir H, Robinson JT, Mesirov JP. Integrative Genomics Viewer (IGV): High-performance genomics data visualization and exploration. Brief Bioinform. 2013;14:178–92. doi:10.1093/bib/bbs017.

10. Lovén J, Hoke HA, Lin CY, Lau A, Orlando DA, Vakoc CR, et al. Selective inhibition of tumor oncogenes by disruption of super-enhancers. Cell. 2013;153:320–34. doi:10.1016/j.cell.2013.03.036.

11. Whyte WA, Orlando DA, Hnisz D, Abraham BJ, Lin CY, Kagey MH, et al. Master transcription factors and mediator establish super-enhancers at key cell identity genes. Cell. 2013;153:307–19. doi:10.1016/j.cell.2013.03.035.

12. Bailey TL, Johnson J, Grant CE, Noble WS. The MEME Suite. Nucleic Acids Res. 2015;43:W39–49.

13. Landt SG, Marinov GK, Kundaje A, Kheradpour P, Pauli F, Batzoglou S, et al. ChIP-seq guidelines and practices of the ENCODE and modENCODE consortia. Genome Research. 2012;22:1813–31. doi:10.1101/gr.136184.111.
