# Supplementary material for: SEAseq: a portable and cloud-based chromatin occupancy analysis suite
Source: BMC Bioinformatics. 2022 Feb 23;23:77. doi: 10.1186/s12859-022-04588-z (PMC8864840; doi:10.1186/s12859-022-04588-z)
Supplement: Supplementary file 3 — Additional file 3. Case study LIN28B. The complete HTML quality statistics report for LIN28B ChIP-seq analysis. [file 12859_2022_4588_MOESM3_ESM.html]

SEAseq Report

|  |  |
| --- | --- |
|  | SEAseq Quality Statistics and Evaluation Report |

| LEGEND |
| --- |
| EXCELLENT |
| GOOD |
| AVERAGE |
| BELOW-AVERAGE |
| POOR |


## Sample FASTQs Quality Results

| Sample Name | Overall Quality | Raw Reads | Read Length | Base Quality | Sequence Diversity | Aligned Percent | NRF | PBC | NSC | RSC |
| --- | --- | --- | --- | --- | --- | --- | --- | --- | --- | --- |
| SRR10259398 | GOOD | 54894988 | 75 | pass | pass | 80.787 | 0.8183 | 0.8378 | 1.0266 | 0.8804 |

## Control FASTQs Quality Results

| Sample Name | Overall Quality | Raw Reads | Read Length | Base Quality | Sequence Diversity | Aligned Percent | NRF | PBC | NSC | RSC |
| --- | --- | --- | --- | --- | --- | --- | --- | --- | --- | --- |
| SRR10259397 | GOOD | 45603300 | 75 | pass | pass | 78.713 | 0.9425 | 0.9713 | 1.0132 | 0.7821 |

## Overall Quality Evaluation and Statistics Results

| DATA | Overall Quality | Raw Reads | Base Quality | Sequence Diversity | Aligned Percent | Estimated Fragment Width | Estimated Tag Length | NRF | PBC | NSC | RSC | FRiP | Total Peaks | Normalized Peaks\* | Linear Stitched Peaks | SE-like Enriched Regions |
| --- | --- | --- | --- | --- | --- | --- | --- | --- | --- | --- | --- | --- | --- | --- | --- | --- |
| SAMPLE | GOOD | 56053120 | pass | pass | 79.118 | 121 | 75 | 0.8183 | 0.8378 | 1.0266 | 0.8804 | 0.0218 | 11248 | 11248 | 540 | 52 |
| CONTROL |  | 46608378 | pass | pass | 77.016 | 200 | 75 | 0.9425 | 0.9713 | 1.0132 | 0.7821 | 0.0015 | 605 |  |  |  |

**\*** Peaks identified after Input/Control correction.

**Abbreviations** (adopted from Landt et al, Genome Res. 2012)

- NRF : Non-Redundant Fraction.
- PBC : PCR BottleNeck Coefficient.
- NSC : Normalized Strand Cross-correlation coefficient.
- RSC : Relative Strand Cross-correlation coefficient.
- FRiP : Fraction of Reads in Peaks.

  
Definitions for all metrics can be found on the SEAseq website.
