# Supplementary material for: SEAseq: a portable and cloud-based chromatin occupancy analysis suite
Source: BMC Bioinformatics. 2022 Feb 23;23:77. doi: 10.1186/s12859-022-04588-z (PMC8864840; doi:10.1186/s12859-022-04588-z)
Supplement: Supplementary file 4 — Additional file 4. SEAseq inputs and outputs. Input files used for SEAseq analysis and detailed descriptions of the Output directories and files generated using SEAseq. [file 12859_2022_4588_MOESM4_ESM.doc]

## **SEAseq Input Files**

SEAseq requires one or more single-end sequenced FASTQ files either as sample FASTQ or Input/Control FASTQ. FASTQ files can be manually uploaded and/or publicly available data can be specified by their Short Read Archive (SRA) identifier (SRR). SRA files are downloaded as FASTQ files using the SRA toolkit [1]. Notably, SEAseq requires single-end data as input, however paired-end data can also be provided but will be processed as single-ended.

In addition, SEAseq requires that the user provides a reference genome FASTA and a gene annotation file (in either GTF, GFF or GFF3). Optional input files accepted by SEAseq include a genome blacklists bed file, as well as one or more position weight matrix databases provided from the MEME Suite.

SEAseq supports Genome reference and Gene annotation files from most genome repositories, such as UCSC, ENSEMBL, RefSeq or GENCODE. Genome blacklists are typically either the Ultra-High Signal (UHS) blacklists, the original ENCODE blacklist or Duke Excluded Regions (DER), or the ENCODE Data Analysis Center (DAC) blacklisted regions [2].

The input files can be in their compressed gzip format where applicable. Table D1 describes the accepted SEAseq inputs.

Table D1. SEAseq Input Fields

| **Name** | **Definition** | **Type** | **Description** | **Example (accepted file/data types)** | **Necessity** |
| --- | --- | --- | --- | --- | --- |
| reference | Genome Reference | File | A genome reference in FASTA format. | **.fa*  **.fasta*  (acceptable in compressed gzip format) | required |
| gtf | Gene Annotation | File | A gene position database file | **.gtf*  **.gff*  **.gff3*  (acceptable in compressed gzip format) | required |
| sample_fastq | Sample FASTQ files | Array of files | One or more Sample FASTQs. | **.gz*  (compressed gzip format required) | optional |
| sample_sraid | Sample SRA run accession identifiers (SRRs) | Array of strings | One or more Sample SRRs. | *SRR123456789* | optional |
| control_fastq | Input/Control FASTQ files | Array of files | One or more Input/IgG/Control FASTQs | **.gz*  (compressed gzip format required) | optional |
| control_sraid | Input/Control SRRs | Array of strings | One or more Input/IgG/Control SRRs. | *SRR123456789* | optional |
| blacklist | Blacklist regions | File | UHS/DER/DAC or custom blacklisted regions file. (acceptable in compressed gzip format) | **.bed*  (acceptable in compressed gzip format) | optional |
| motif_databases | Motif databases | Array of files | One or more position weight matrix database files | **.meme* | optional |
| bowtie_index | Genome Bowtie indexes | Array of files | The set of six genome bowtie v1 index files. | **.ebwt* | optional |
| results_name | Results custom name | String | Preferred analysis results name. (recommended if multiple FASTQs are provided) | *The-results* | optional |
| output_directory* | SEAseq output directory | String | The name of the output directory. | *OUTPUT* | optional |

* Not available in SEAseq Cloud. Rather the output directory should be specified in the “**Analysis Settings**” 🡪 “**Execution Output Folder**” field in the SEAseq “**Run Analysis**” Page.

More information on input files and configuration are available at the [SEAseq documentation page](https://github.com/stjude/seaseq/#inputs).

## **SEAseq Output Directories**

All analysis results files and folders will be saved into the specified output directory. To allow of easy exploration of results files, SEAseq organizes the results files into descriptive sub-directories. For the different analyses SEAseq performs, the multiple output files are grouped into the following seven major directories:

Multiple FASTQs are

- ***BAM_Density*** – contains reads coverage density distributions in both promoters and genic regions. The profiling matrices are extrapolated from the gene annotation file provided as input. The files generated are:
  1. The distribution matrices in promoters, upstream, downstream and the genebody regions.
  2. Average density distribution plots in high resolution pdf and png formats.
  3. Density heatmaps in high resolution pdf and png formats.
  4. A customizable Rscript for editing and re-creation of provided plots.
- ***BAM_files*** – contains all generated mapping files. The files provided will have the following filenames:
  1. ***.sorted.bam**: the alignments sorted by chromosomal coordinates.
  2. ***.rmdup.bam**: the alignments after removing duplicates.
  3. ***.bklist.bam**: the alignments after exclusion of optionally provided blacklist regions.
- ***COVERAGE_files*** – contains normalized signal data tracks of both the Narrow and Broad peaks identified for easy visualization across most genome browsers available, such as GenomePaint[3], the UCSC genome browser[4] (in wiggle [**.wig**] and bigwig [**.bw**] formats) or IGV[5] (in tdf [**.tdf**] format.)
- ***MOTIFS*** – contains the files generated from motifs discovery and prediction analysis using the AME and MEME-chip tools from the MEME Suite[6]. Motif discovery and enrichment analysis are performed on both peaks and peak summits.
- ***PEAKS*** – has all the peak files sub grouped into:
  1. ***NARROW_peaks***: for shorter or narrow regions of enrichment using MACS, which is recommended for profiling many transcription factors. SEAseq performs three different peak calls using MACS v1.4.2 [7].
     - **Peaks identified excluding duplicate tags** (keep-dup=auto): The peaks identified excluding duplicate tags preventing erroneous signal calls from noise. (Files will be named as *<samplename>-p9_kd-auto*)
     - **Peaks identified keeping duplicate tags** (keep-dup=all): The peaks are identified using duplicates to estimate signal, this will be used to call linear-stitched (or Enhancers) peaks and SE-like stitched (or Super-Enhancers) peaks using the ROSE program. (Files named as *<samplename>-p9_kd-all*)
     - **Peaks identified using a defined shift size** (shiftsize=200): This is designed to generate an unbiased signal coverage plot, which may be used for comparison with multiple samples purposes. (Files named as *<samplename>-nm*)
  2. ***BROAD_peaks***: for broad peaks or broad domains using SICER[8], which is recommended for profiling some histone modifications, including H3K27me3.
  3. ***STITCHED_peaks***: for clusters of stitched peaks identified using the ROSE program [9, 10].
- ***PEAKS_Annotation*** – consists of the peak-to-gene annotation files and quantification of peak occupancy plots for broad and narrow peaks in genic regions. The files description are:
  1. **centerofpeaks_closest.regions.txt**: comprises of genes with TSS near the center of peaks.
  2. **peaks_within_genebody.regions.txt**: comprises of peaks overlapping gene regions.
  3. **peaks_within_promoter.regions.txt**: contains peaks overlapping promoters.
  4. **peaks_within_window.regions.txt**: contains peaks overlapping windows.
  5. **peaks_compared_regions.peaks.txt**: contains peaks identified in previous overlapping regions and comparison of all regions.
  6. **peaks_compared_regions.genes.txt**: consist of all genes identified in previous overlapping regions and comparison of all regions.
  7. **peaks_compared_regions.distribution.pdf**: comprises of bar plots showing percentage distribution of peaks in genic regions.
- ***QC*** – includes the FastQC reports and the SEAseq quality metrics results as a tab-delimited file (.txt), and color flagged HTML file (.html).

## **References**

1. Leinonen R, Sugawara H, Shumway M, Collaboration on behalf of the INSD. The Sequence Read Archive. Nucleic Acids Res. 2011;39 suppl_1:D19–21. doi:10.1093/NAR/GKQ1019.

2. Amemiya HM, Kundaje A, Boyle AP. The ENCODE Blacklist: Identification of Problematic Regions of the Genome. Sci Rep. 2019;9:9354. doi:10.1038/s41598-019-45839-z.

3. Zhou X, Wang J, Patel J, Valentine M, Shao Y, Newman S, et al. Exploration of Coding and Non-coding Variants in Cancer Using GenomePaint. Cancer Cell. 2021;39:83-95.e4.

4. Kuhn RM, Haussler D, James Kent W. The UCSC genome browser and associated tools. Brief Bioinform. 2013;14:144–61. doi:10.1093/bib/bbs038.

5. Thorvaldsdóttir H, Robinson JT, Mesirov JP. Integrative Genomics Viewer (IGV): High-performance genomics data visualization and exploration. Brief Bioinform. 2013;14:178–92. doi:10.1093/bib/bbs017.

6. Bailey TL, Johnson J, Grant CE, Noble WS. The MEME Suite. Nucleic Acids Res. 2015;43:W39–49.

7. Zhang Y, Liu T, Meyer CA, Eeckhoute J, Johnson DS, Bernstein BE, et al. Model-based analysis of ChIP-Seq (MACS). Genome Biol. 2008;9. doi:10.1186/gb-2008-9-9-r137.

8. Zang C, Schones DE, Zeng C, Cui K, Zhao K, Peng W. A clustering approach for identification of enriched domains from histone modification ChIP-Seq data. Bioinformatics. 2009;25:1952–8. doi:10.1093/bioinformatics/btp340.

9. Lovén J, Hoke HA, Lin CY, Lau A, Orlando DA, Vakoc CR, et al. Selective inhibition of tumor oncogenes by disruption of super-enhancers. Cell. 2013;153:320–34. doi:10.1016/j.cell.2013.03.036.

10. Whyte WA, Orlando DA, Hnisz D, Abraham BJ, Lin CY, Kagey MH, et al. Master transcription factors and mediator establish super-enhancers at key cell identity genes. Cell. 2013;153:307–19. doi:10.1016/j.cell.2013.03.035.
