# Supplementary material for: SEAseq: a portable and cloud-based chromatin occupancy analysis suite
Source: BMC Bioinformatics. 2022 Feb 23;23:77. doi: 10.1186/s12859-022-04588-z (PMC8864840; doi:10.1186/s12859-022-04588-z)
Supplement: Supplementary file 5 — Additional file 5. Case study datasets. Case Study Datasets and Genome Files information. [file 12859_2022_4588_MOESM5_ESM.doc]

## **Case Study Datasets and Genome Files Used.**

The previously published datasets used to showcase our application.

1. **GSE138742 by Tao,T. *et al.* (2020) LIN28B regulates transcription and potentiates MYCN-induced neuroblastoma through binding to ZNF143 at target gene promotors. *Proc. Natl. Acad. Sci. U. S. A.*, 117, 16516–16526.**

All datasets can be downloaded from SRA under accession number SRP225129 at https://www.ncbi.nlm.nih.gov/sra/SRP225129.

We individually analyzed the FASTQ files of LIN28B, ZNF143 and doxycycline-inducible engineered LIN28B by providing their SRA run identifiers (Table C1).

Table C1. Tao *et al.*, 2020 ChIP-seq FASTQs data accession numbers.

| **Cell Line** | **ChIP-seq antibody** | **GEO** | **SRR** |
| --- | --- | --- | --- |
| BE2C | Input | GSM4118359 | SRR10259397 |
| BE2C | LIN28B | GSM4118360 | SRR10259398 |
| BE2C | ZNF143 | GSM4118362 | SRR10259400 |
| BE2C | yDox-Input | GSM4118367 | SRR10259405 |
| BE2C | yDox-LIN28B | GSM4118368 | SRR10259406 |

The genome reference information used are:

- - Human hg19 reference genome from GENCODE Release 19 (GRCh37.p13): <http://ftp.ebi.ac.uk/pub/databases/gencode/Gencode_human/release_19/GRCh37.p13.genome.fa.gz>. We excluded scaffolds, assembly patches and haplotypes.

$ # Syntax:

$ wget http://ftp.ebi.ac.uk/pub/databases/gencode/Gencode_human/release_19/GRCh37.p13.genome.fa.gz

$ gunzip GRCh37.p13.genome.fa.gz

$

$ # selecting only reference chromosomes

$ head -n 50 GRCh37.p13.genome.fa > hg19_chroms.fa

$ gzip hg19_chroms.fa

- - Human hg19 GTF comprehesive gene annotation from GENCODE Release 19 (GRCh37.p13): <http://ftp.ebi.ac.uk/pub/databases/gencode/Gencode_human/release_19/gencode.v19.annotation.gtf.gz>.
  - UHS blacklists from [hg19-blacklist.v2.bed.gz](https://github.com/Boyle-Lab/Blacklist/raw/master/lists/hg19-blacklist.v2.bed.gz).
  - Position Weight Matrices (PWM) were downloaded from the MEME Suite Motif Database page (<https://meme-suite.org/meme/meme-software/Databases/motifs/motif_databases.12.21.tgz>). The PWM used are:
    1. HOCOMOCOv11: HUMAN/HOCOMOCOv11_full_HUMAN_mono_meme_format.meme (Kulakovski*y et a*l., 2018).
    2. JASPAR2018: JASPAR/JASPAR2018_CORE_vertebrates_redundant.meme (Kha*n et a*l., 2018).
    3. CisBP: CIS-BP_2.00/Homo_sapiens.meme (Weirauc*h et a*l., 2014).
    4. Jolma2013: EUKARYOTE/jolma2013.meme (Jolm*a et a*l., 2013).

1. **GSE31558 by Botcheva,K. *et al.* (2011) Distinct p53 genomic binding patterns in normal and cancer-derived human cells. *Cell Cycle*, 10, 4237–4249.**

All dataset can be downloaded from SRA under accession number SRP007953 at https://www.ncbi.nlm.nih.gov/sra/SRP007953.

We executed SEAseq on the ChIP-seq FASTQ files (both input and p53_ChIP-seq) by also providing their SRA run identifiers (Table C2).

Table C2. Botcheva *et al.*, 2011 ChIP-seq FASTQs data accession numbers.

| **Cell Line** | **ChIP-seq antibody** | **GEO** | **SRR** |
| --- | --- | --- | --- |
| IMR90 | p53 | GSM783262 | SRR333829 |
| IMR90 | Input | GSM783263 | SRR333830 |

To ensure comparable replication of analysis, we uploaded curated hg18 genome data files, which are:

- - Human hg18 reference genome from UCSC ftp database (<https://hgdownload.soe.ucsc.edu/goldenPath/hg18/bigZips/chromFa.zip>). We excluded unplaced and haploid sequences from our merged genome file

$ # Syntax:

$ wget https://hgdownload.soe.ucsc.edu/goldenPath/hg18/bigZips/chromFa.zip;

$ unzip chromFa.zip -d chromFA; cd chromFA;

$ for chrom in $(ls -1 | grep -v "_random" | grep -v "_hap"); do cat $chrom >>hg18.fa; done;

$ gzip hg18.fa

- - We created the hg18 gene annotation file (.gtf) from the UCSC Table database using the UCSC genePredToGtf program (available to download from <https://hgdownload.cse.ucsc.edu/admin/exe/>).

Based on our system requirements, we downloaded the mac OSX version (<http://hgdownload.cse.ucsc.edu/admin/exe/macOSX.x86_64/genePredToGtf>).

$ # Syntax used

$ echo -e 'db.host=genome-mysql.cse.ucsc.edu\ndb.user=genomep\ndb.password=password' > ~/.hg.conf

$ chmod 600 ~/.hg.conf

$ chmod +x genePredToGtf

$ ./genePredToGtf hg18 refGene hg18.gtf

$ gzip hg18.gtf
